# Linezolid-Resistant *Enterococcus* spp. Isolates from Foods of Animal Origin—The Genetic Basis of Acquired Resistance

**DOI:** 10.3390/foods11070975

**Published:** 2022-03-28

**Authors:** Urszula Zarzecka, Arkadiusz Józef Zakrzewski, Wioleta Chajęcka-Wierzchowska, Anna Zadernowska

**Affiliations:** Department of Industrial and Food Microbiology, Faculty of Food Science, University of Warmia and Mazury in Olsztyn, Plac Cieszyński 1, 10-726 Olsztyn, Poland; urszula.zarzecka@uwm.edu.pl (U.Z.); arkadiusz.zakrzewski@uwm.edu.pl (A.J.Z.); anna.zadernowska@uwm.edu.pl (A.Z.)

**Keywords:** linezolid resistant enterococci, food, *cfr*, *optr*A, *poxt*A, antibiotic resistance, resistance genes

## Abstract

Enterococci are important opportunistic pathogens with the capacity to acquire and spread antibiotic resistance. At present, linezolid-resistant enterococci (LRE) pose a great challenge. Linezolid is considered as a last resort antibiotic in the treatment of enterococcal infections, so it is important to monitor the occurrence of LRE in various environments. The aim of this study was to define the genetic mechanisms of linezolid resistance in enterococci (*E. faecalis*, *E. faecium*, *E. hirae*, *E. casseliflavus*) isolated from foods of animal origin (*n* = 104). Linezolid resistance (LR) was shown by 26.9% of isolates. All of them displayed linezolid MICs of 8–32 µg/mL, and 96.4% of them were multidrug multidrug-resistant. The most common acquired linezolid resistance gene in LR isolates was *poxtA* (64%), followed by *optrA* (28%) and *cfr* (12%). According to the authors’ knowledge, this research is the first to indicate the presence of the *cfr* gene among isolates from food. In 28.6% of the isolates, the point mutation G2576T in the V domain of the 23S rRNA was responsible for linezolid resistance. All isolates harbored the wild-type *rplC*, *rplD* and *rplV* genes. The obtained results indicate that linezolid resistance among enterococci in animal-derived food may result from various genetic mechanisms. The most worrying is that this resistance is encoded on mobile genetic elements, so there is a risk of its rapid transmission, even despite the lack of selective pressure resulting from the use of antibiotics.

## 1. Introduction

Enterococci are an important part of the commensal microbiota in the gastrointestinal tract of warm-blooded animals. Therefore, these microorganisms are common in foods of animal origin due to contamination with intestinal contents, which is associated with the risk of bacterial cells spreading through the food chain. In addition, microorganisms can enter food from the production environment [1,2,3]. Enterococci are able to survive adverse environmental conditions and quickly adapt to changing conditions. This allows them to survive the food production and storage conditions, so they are very often residual microflora of ready-to-eat foods [4,5]. Although these microorganisms were considered non-pathogenic for many years, the incidence of enterococcal infections has recently increased rapidly. This has made them a subject of increased interest. In the last few decades, enterococci have become pathogens of higher importance [6,7]. They are intrinsically resistant to many antibiotics, i.e., cephalosporins, low-level of aminoglycosides and polymyxins, and are also able to acquire resistance to β-lactams, high-level of aminoglycosides, glycopeptides, macrolides and tetracyclines, mainly by acquiring plasmids or transposons carrying resistance genes. Enterococci also are able to transfer antibiotic resistance determinants to other microorganisms, including pathogens [7,8,9].

In 2000, linezolid became the first oxazolidinone antibiotic accepted for application into clinical use by the US Food and Drug Administration (FDA). During the last 40 years, oxazolidinones have been considered a truly new class of antibiotics, which are currently used in clinics. In Poland, Linezolid was approved to use in 2002. It is highly efficient against Gram-positive pathogens, such as methicillin-resistant *Staphylococcus aureus* (MRSA) and vancomycin-resistant *Enterococcus* (VRE). Its mechanism of action is based on the inhibition of protein synthesis by interaction with domain V of the 23S ribosomal RNA (rRNA) [10]. In recent years, the frequency of the occurrence of linezolid-resistant *Enterococcus* strains has increased [11]. Linezolid resistance in *Enterococcus* spp. is most often associated with mutations in the domain V of the 23S rRNA and ribosomal proteins L3, L4 and L22 [11,12].

The first reported transferable linezolid resistance gene was the plasmid-mediated gene *cfr* encoding methyltransferase. The resistance phenotype mediated by this gene is referred to a PhLOPSA, which is an acronym for resistance to the following antibiotics: lincosamides, phenicols, oxazolidinones, streptogramin A and pleuromutilin [13]. Moreover, the transfer of the *cfr* gene has been demonstrated across various bacterial species and genera [14,15]. Regarding *Enterococcus*, the *cfr* gene was reported for the first time in *E. faecalis* of animal origin. In further studies in enterococci, several various conjugative plasmids carrying this gene were identified [16]. *E. faecium* isolates with the *cfr* gene are also increasingly being reported [17]. Since the *cfr* gene was first identified in a bovine *Staphylococcus* isolate in 2000, this gene has been detected in *Enterococcus* isolated from humans and animals, such as pigs, cattle, horses and poultry. LaMarre et al. [18] suggested that the fitness cost associated with the carriage of *cfr* is low, and it may contribute to the spread of this gene.

In 2015, a novel transferable oxazolidone-resistance gene (*optr*AIt) was identified in *Enterococcus* spp., which encodes an ATP-binding cassette (ABC)-F protein, conferring cross-resistance to oxazolidinones and phenicols and mediating resistance through target protection [19,20,21]. This is a transferable gene located on plasmids transferring resistance to other important antibiotics: macrolides, lincosamides, streptogramin B, aminoglycosides and phenicols. It was detected for the first time in *E. faecalis* of human origin, and further studies have reported the presence of the *optr*A in *E. faecium* isolates [22,23,24,25,26]. According to surveillance studies, the *optr*A gene is more frequent in enterococci isolated from livestock than from humans [27]. The occurrence of the *optr*A gene was detected in the *E. faecalis* strain isolated from veal meat samples (in 2015) and in the *E. faecium* strain isolated from turkey meat (in 2012) [28].

Recently, a novel gene (*poxt*A) conferring resistance to oxazolidinones was found in MRSA and enterococci. The prevalence of *poxt*A among enterococci is currently under investigation [29].

The aim of the current study was to determine the incidence of linezolid-resistant *Enterococcus* (LRE) strains in foods of animal origin and to determine the genetic basis of this resistance by analyzing the occurrence of the *optr*A, *poxt*A and *cfr* genes.

## 2. Materials and Methods

### 2.1. Sampling

Two hundred eighty samples of foods of animal origin included both raw and ready-to-eat food: raw milk (*n* = 50), raw fish and shrimps (*n* = 200) and sushi (*n* = 30) were collected from farms in northern Poland and from markets and restaurants in Olsztyn, Poland. Immediately after purchasing, the samples were transported to a laboratory in a cold chain and analyzed within 4 h from purchase.

### 2.2. Isolation and Preliminary Identification

The isolation and identification of the strains was carried out according to the methodology described previously [2]. The first step was preliminary propagation of 10 g or 10 mL of the sample in 90 mL of buffered peptone water (Merck, Darmstadt, Germany). After incubation at 37 °C for 24 h the full loop of cultures was inoculated on Slanetz–Bartley agar (Merck, Darmstadt, Germany) and incubated at 37 °C for 48 h. Then, all colonies with enterococcal morphology were subjected to Gram staining and an assay for catalase and oxidase production. Until further analyses were carried out, the strains were stored at −80 °C using the Microbank system (Biocorp., Warszawa, Poland).

### 2.3. Identification of Enterococcus Isolates

Isolates identification was performed with the MALDI-TOF MS (matrix-assisted laser desorption/ionization time-of-flight mass spectrometry) technique using a VITEK^®^ MS mass spectrometer (bioMérieux, Durham, NC, USA), as described previously [2]. Isolates were first streaked on Tryptic Soy Agar (TSA) (Merck, Darmstadt, Germany) and incubated overnight in 37 °C. Single colonies were applied to spots of the test slide, overlaid with 0.5 µL of formic acid (FA) and 1 µL of matrix solution (α-cyano-4-hydroxycinnamic acid, CHCA) and air-dried. *Escherichia coli* ATCC 8739 was used as a reference strain to validate the protocol. Measures were taken in triplicate. Obtained spectra were compared against the VITEK MS SARAMIS research-use-only (RUO) database. Identification was considered definitive when the probability was <90%.

### 2.4. Antimicrobial Resistance

Antibiograms were performed by the Kirby–Bauer method on the Mueller–Hinton agar (Merck, Darmstadt, Germany). Suspensions in sterile normal saline solution (0.89% NaCl) with a density of 0.5 McFarland were prepared using bacterial cultures on TSA agar (Merck, Darmstadt, Germany). Eighteen antibiotics recommended for use in the treatment of clinical infections or in agricultural procedures were chosen for analysis: ampicillin 10 µg (AMP), norfloxacin 10 µg (NOR), ciprofloxacin 5 µg (CIP), gentamicin 120 µg (CN), teicoplanin 30 µg (TEC), vancomycin 30 µg (VAN), erythromycin 15 µg (E), streptomycin 300 µg (S), quinupristin/dalfopristin 15 µg (QD), tigecycline 30 µg (TGC), tetracycline 30 µg (TE), doxycycline 30 µg (DO) minocycline 30 µg, fosfomycin 200 µg (FOT), chloramphenicol 30 µg (C), rifampin 5 µg (RD), nitrofurantoin 300 µg (F) and linezolid 30 µg (LZD) (Oxoid, Poznan, Poland). *E. faecalis* strains were not studied for quinupristin/dalfopristin resistance, because they have intrinsic resistance to this antibiotic. After incubation, growth inhibition zones were measured and referred to the guidelines of the CLSI-Clinical and Laboratory Standards Institute [30]. As quality control organisms, *E. faecalis* ATCC 29212 and *S. aureus* ATCC 25923 were used.

The second step was determination of the minimum inhibitory concentration (MIC) for linezolid with the agar dilution method with the Mueller–Hinton agar (Merck, Darmstadt, Germany). The gradient strips (MIC Test Strip) were used for the analysis (Liofilchem, Roseto Degli Abruzzi, Teramo, Italy). The obtained results were compared in accordance with the recommendations of the CLSI (CLSI, 2019). The MIC breakpoint value for linezolid was ≥8 μg/mL.

### 2.5. Genomic DNA Isolation and Detection of Linezolid Resistance Mechanisms

Molecular analyses were performed for each isolate phenotypically resistant for linezolid. For genomic DNA extraction, the colonies on TSA (Merck, Darmstadt, Germany) were suspended in 100 µL of Tris-EDTA buffer (Sigma-Aldrich, Poznań, Poland) and lysed by the lysozyme (0.6 mg/mL) (A&A Biotechnology, Gdańsk, Poland). The total genomic DNA extraction was carried out using the Genomic Mini DNA isolation and purification kit (A&A Biotechnology, Gdańsk, Poland), according to the manufacturer’s instructions. The obtained DNA was suspended in 200 μL of Tris-HCl buffer (10 mM, pH 8.5) and stored at −20 °C. DNA samples were matrixed for the PCR reaction carried out in the study.

To determine the linezolid resistance mechanism, each strain was tested for mutations in the domain V of the 23S rRNA and ribosomal proteins L3, L4 and L22. The occurrence of acquired linezolid resistance genes *optr*A, *poxt*A and *cfr* was also checked.

The genes encoding ribosomal proteins *rp*lV (L22) *rp*lC (L3), *rplD* (L4) and 23S rRNA were amplified with primers and annealing temperatures according to Mališová et al. (Table 1) [31]. The PCR products were cleaned with a Clean-Up purification kit (A&A Biotechnology, Gdańsk, Poland) and then sequenced by a commercial enterprise (Genomed, Warsaw, Poland). The obtained sequences were compared to the wild-type sequences from *E. faecium* DO (CP003583.1), *E. faecalis* ATCC 29212 (CP008816.1) and *E. avium* ATCC 14025 (ASWL01000001.1) strains.

The presence of acquired genes encoding resistance to linezolid was determined according to the method proposed by Bender et al. [32] using primers listed in Table 1. *Enterococcus faecalis* 1474/13 and *Staphylococcus aureus* 5093/08 from the collection of Polish National Medicines Institute were used as positive controls.

All of the amplicons were evaluated by 1.5% agarose gel electrophoresis in TBE buffer followed by staining in ethidium bromide (0.5 mg/mL). Products were visualized under ultraviolet light by using the G-box system (Syngene).

**Table 1:** Oligonucleotides used in the study.

Gene	Primer Sequence 5′-3′	Annealing Temperature	References
23S rRNA	F: GTAACGATTTGGGCACTGTCG R: CGATTAGTATTGGTCCGCTC	55 °C	[31]
*rpl*C	F: GCGCTTCATTCGTGAATTCAA R: TTCTTTCTGCATCGACACGTACAA	50 °C	
*rpl*D	F: ACGATGCAATCGTAATGCAA R: TTCAGCAACTTTTTCTGACAA	51 °C	
*rpl*V	F: GGACATGCTGCTGACGATA R: ACCATTTAGCATCCCAGTCG	50 °C	
*cfr*	F: TGAAGTATAAAGCAGGTTGGGAGTCA R: ACCATATAATTGACCACAAGCAGC	50 °C	[33]
*optr*A	F: TACTTGATGAACCTACTAACCA R: CCTTGAACTACTGATTCTCGG	50 °C	[21]
*poxt*A	F: AAAGCTACCCATAAAATATC R: TCATCAAGCTGTTCGAGTTC	50 °C	[32]

## 3. Results

### 3.1. Isolation and Identification of Enterococci

From 280 food samples, a total number of 104 strains belonging to the genus *Enterococcus* were isolated. Their identification confirmed that the strains belonged to four species: *E. faecalis* (74.0%, *n* = 77), *E. faecium* (12.5%, *n* = 13), *E. casseliflavus* (4.8%, *n* = 5) and *E. hirae* (3.9%, *n* = 4). There were also five strains not identified to the species level.

### 3.2. Antibiotic Resistance Patterns of Enterococcus Isolates

Phenotypic linezolid resistance was shown by 28 strains. The majority (89.3%, *n* = 25) belonged to *E. faecalis* species, two strains (7.1%) were identified as *E. faecium* and one strain (3.6%) was identified as *E. hirae*. All of the linezolid-resistant (LZD^R^) isolates displayed linezolid MICs of 8–32 µg/mL, and 96.4% of them were resistant to antibiotics from three or more classes, mainly to ansamycins, tetracyclines and macrolides (Table 2).

Due to the different number of analyzed strains belonging to individual species, no reliable statistical analysis could be performed indicating differences in the frequency of linezolid resistance among various *Enterococcus* species.

### 3.3. Detection of Linezolid Resistance Mechanisms

Phenotypic linezolid resistance was demonstrated by 28 strains that were subjected to genotypic analysis to determine the genetic basis and mechanism of resistance.

In eight strains (28.6%), the point mutation G2576T in the V domain of the 23S rRNA was responsible for linezolid resistance. All isolates harbored the wild-type and *rpl*V *rpl*C and *rpl*D gene (GenBank accession no. ON032309; ON032310; ON032311; ON032312; ON032313; ON032314; ON032315; ON032316).

**Figure 1:**
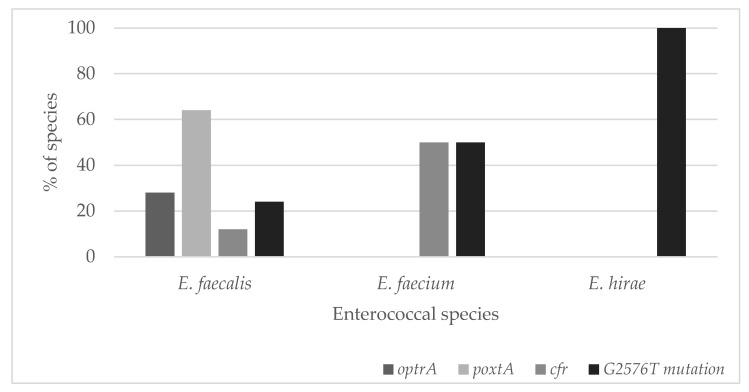
The genetic background of linezolid-resistant (LZD^R^) *Enterococcus* strains analyzed in this study.

The most common linezolid resistance gene was the *poxt*A gene, which was present in 64% of *E. faecalis* strains (*n* = 16). Its presence was not found in strains from other species. Similar to the occurrence of the *optr*A gene, it was present only in *E. faecalis* strains (28%, *n* = 7). The *cfr* gene occurred in 12% of *E. faecalis* strains (*n* = 3) and 50% of *E. faecium* strains (*n* = 1). (Figure 1).

Since the number of strains from various species was uneven, no relationship could be demonstrated for these three genes depending on the species. The obtained characteristics of all LZD^R^ strains are summarized in Table 1.

## 4. Discussion

Linezolid is an antibiotic normally used in the treatment of important infections caused by multidrug-resistant Gram-positive bacteria. Currently, linezolid is considered as a last-resort antibiotic, which means the antibiotic which is used after all other antibiotic options have failed to produce an adequate response. Since linezolid was introduced into clinical use, the number of reported linezolid-resistant enterococci (LRE) is still increasing [33,34]. For several years, they have been increasingly appearing among clinical isolates and recently also in food [35,36,37]. However, there is very little research in the literature determining the genotype of food-derived isolates, and the current study is one of the first.

In the current study, 26.9% of isolates were resistant to linezolid, and they displayed linezolid MICs of 8–32 µg/mL. Most of them belonged to the *E. faecalis* species. These strains in majority were multidrug resistant. In our study, 10 linezolid-resistant isolates were also vancomycin-resistant. Although the occurrence frequency of linezolid resistance is moderate, it has been reported that the occurrence and dissemination of linezolid-resistant enterococci may result in limited therapeutic options for treatment of enterococcal infections. However, other studies have reported a lower percentage of LRE isolated from humans and animals [27,38,39]. The results obtained in this study are reflected in other studies on enterococci isolated from foods of animal origin, irrespective of the region [9,10]. Some authors have found a higher percentage of strains resistant to linezolid among the isolates tested. On the other hand, the analysis of resistance to antibiotics of enterococci from foods of plant origin showed a lower percentage of strains resistant to linezolid [9]. This suggests that resistance to linezolid may be due to the selective pressure associated with the use of antibiotics in animal husbandry.

Point mutations in the V domain of 23S rRNA and genes encoding ribosomal proteins L22 (*rpl*V), L3 (*rpl*C) and L4 (*rpl*D) are considered to be the most common mechanisms of resistance to linezolid [40]. This was not confirmed in the current study, which found that only 28.6% of linezolid-resistant enterococci’s resistance resulted from the mutation G2576T in the 23S rRNA. Mutations in *rpl*C, *rpl*D and *rpl*V genes were also not detected.

According to the literature, mutations in *rpl*C, *rpl*D and *rpl*V genes occurred more commonly in coagulase-negative staphylococci (CoNS) than in enterococci [41].

In the present study, the *poxt*A gene was the main gene encoding for linezolid resistance. Its prevalence in enterococci from food-producing animals and foods of animal origin is a challenge to public health. It has been proven that they might be passed on to humans through the consumption of food [42]. Antonelli et al. [29] reported that the uncontrolled use of oxazolidinones in food-producing animals could cause the spread of strains carrying the *poxt*A gene. Thus, the spread of the *poxt*A gene among enterococci from humans and food-producing animals requires increased scrutiny [43].

Seven strains isolated in this study carried the *optr*A gene. It is noteworthy that this gene could be horizontally transferred among enterococci [24,44]. However, in previous studies, the *optr*A gene was found more frequently in foods of animal origin than in this study [27,39]. In addition, this gene was the most commonly found among *E. faecalis* strains [39]. What is interesting is that the *optr*A gene was detected in an *E. faecium* isolate for the first time two years before the first linezolid applications in China [23]. Although the current study also showed the co-occurrence of *poxt*A and *optr*A genes in some *E. faecalis* isolates, the co-occurrence of *cfr* and *optr*A genes was more commonly found than reported in the literature [42].

Several researchers have pointed to the increasing threat to public health of the occurrence of the *cfr* gene [16]. Zhang et al. suggested that the occurrence of the *cfr* gene among LRE might result in a spreading of linezolid resistance in the environment [45]. It is noteworthy that the current study seems to be the first report indicating the occurrence of the *cfr* gene in isolates from foods of animal origin.

Eight strains did not carry any of these three genes. This may suggest that resistance to linezolid in these strains was due to mutations. Alonso et al. [46] reported that a high MIC value for linezolid is most frequently related to mutations. Smith et al. [47] indicate that the long-term use of this antibiotic may contribute to mutations, resulting in reduced susceptibility to linezolid.

## 5. Conclusions

The current results demonstrated the presence of linezolid-resistant enterococci in foods of animal origin. This resistance may result from various genetic mechanisms. Linezolid is considered to be a last-resort antibiotic in the treatment of enterococcal infections, so it is necessary to monitor the occurrence of linezolid resistance in enterococci isolated from different environments. The most worrying is that this resistance is recorded on mobile genetic elements, so there is a risk of its rapid transmission, even despite the lack of selective pressure in an environment associated with the use of oxazolidinones.

## Figures and Tables

**Table 2:** Characteristics of linezolid-resistant (LZD^R^) strains analyzed in this study.

Species	Antibiotic Resistance Profile	LZD MIC Range [µg/mL]	LZD^R^ Mechanism	Isolation Source (no. of Isolates)
*E. faecalis*	E, TE, LZD, RD	8–32	*poxt*A, *optr*A	Sushi (3)
	NOR, E, TE, LZD, RD	8	*poxt*A, *optr*A	Sushi (3)
	AMP, CIP, NOR, E, FOT, LZD, F, RD	16	*optr*A	Raw milk (1)
	TE, LZD, RD, S	8	*cfr*	Raw milk (1)
	CIP, TE, LZD, RD, DO, MH	8	∆G2576T	Raw milk (1)
	CIP, NOR, VA, LZD, RD	16	*poxt*A	Raw milk (1)
	CIP, LZD, RD	8–16	*poxt*A	Sushi (2)
	VA, LZD, F, RD	8	*poxt*A	Sushi (1)
	CIP, NOR, VA, E, LZD, RD	8	*poxt*A, *cfr*	Sushi (1)
	CIP, VA, LZD, F, RD, C, S	8	*poxt*A	Sushi (1)
	LZD, RD	8–16	*poxt*A	Raw milk (2)
	CIP, VA, E, LZD, F, RD, C	32	∆G2576T	Sushi (1)
	CIP, NOR, VA, E, TE, LZD, RD	16	*cfr*	Raw milk (1)
	CIP, NOR, LZD, RD, MH	8	∆G2576T	Raw milk (1)
	CIP, VA, LZD, RD, C, MH	8	*poxt*A	Sushi (2)
	CIP, NOR, VA, LZD, RD	8	∆G2576T	Sushi (1)
	CIP, NOR, VA, LZD, F, RD, C	32	∆G2576T	Sushi (1)
	CIP, LZD, RD	8	∆G2576T	Sushi (1)
*E. faecium*	CIP, QD, LZD, RD	32	*cfr*	Raw milk (1)
	AMP, CIP, NOR, VA, QD, LZD, RD	16	∆G2576T	Raw milk (1)
*E. hirae*	QD, LZD	8	∆G2576T	Shrimp (1)

Abbrevations: LZD^R^—linezolid resistance, E—erythromycin, TE—tetracycline, LZD—linezolid, RD—rifampin, NOR—norfloxacin, AMP—ampicillin, CIP—ciprofloxacin, FOT—fosfomycin, F—nitrofurantoin, S—streptomycin, DO—doxycycline, MH—minocycline, VA—vancomycin, C—chloramphenicol, QD—quinupristin/dalfopristin. ∆G2576T-mutation G2576T in the 23S rRNA

## Data Availability

Data is contained within the article.

## References

[1] Ogier J.C., Serror P. (2008). Safety assessment of dairy microorganisms: The *Enterococcus* genus. Int. J. Food. Microbiol..

[2] Chajęcka-Wierzchowska W., Zarzecka U., Zadernowska A. (2021). Enterococci isolated from plant-derived food—Analysis of antibiotic resistance and the occurrence of resistance genes. LWT Food Sci. Tech..

[3] Zarzecka U., Zadernowska A., Chajęcka-Wierzchowska W. (2022). Effects of osmotic and high-pressure stress on expression of virulence factors among *Enterococcus* spp. isolated from food of animal origin. Food Microbiol..

[4] Hanchi H., Mottawea W., Sebei K., Hammami R. (2018). The Genus *Enterococcus*: Between Probiotic Potential and Safety Concerns-An Update. Front. Microbiol..

[5] Khan H.A., Ahma A., Mehboob R. (2015). Nosocomial infections and their control strategies. Asian. Pac. J. Trop. Biomedi..

[6] Chajęcka-Wierzchowska W., Zadernowska A., Łaniewska-Trokenheim Ł. (2016). Virulence factors, antimicrobial resistance and biofilm formation in *Enterococcus* spp. isolated from retail shrimps. LWT Food Sci. Tech..

[7] Belgacem Z.B., Abriouel H., Omar N.B., Lucas R., Martínez-Canamero M., Gálvez A., Manai M. (2010). Antimicrobial activity, safety aspects, and some technological properties of bacteriocinogenic *Enterococcus faecium* from artisanal Tunisian fermented meat. Food Cont..

[8] Hammerum A.M., Lester C.H., Heuer O.E. (2010). Antimicrobial-Resistant Enterococci in Animals and Meat: A Human Health Hazard?. Foodborne Pathog. Dis..

[9] Chajęcka-Wierzchowska W., Zadernowska A., Łaniewska-Trokenheim Ł. (2016). Diversity of Antibiotic Resistance Genes in *Enterococcus* Strains Isolated from Ready-to-Eat Meat Products. J. Food Sci..

[10] Na S.H., Moon D.C., Choi M.J., Oh S.J., Jung D.Y., Kang H.Y., Hyun B.H., Lim S.K. (2019). Detection of oxazolidinone and phenicol resistant enterococcal isolates from duck feces and carcasses. Int. J. Food Microbiol..

[11] Hashemian S., Farhadi T., Ganjparvar M. (2018). Linezolid: A review of its properties, function, and use in critical care. Drug Des. Devel. Ther..

[12] Aoki H., Ke L., Poppe S.M., Poel T.J., Weaver E.A., Gadwood R.C., Thomas R.C., Shinabarger D.L., Ganoza M.C. (2002). Oxazolidinone antibiotics target the P site on *Escherichia coli* ribosomes. Antimicrob. Agents Chemother..

[13] Long K.S., Vester B. (2012). Resistance to linezolid caused by modifications at its binding site on the ribosome. Antimicrob. Agents Chemother..

[14] Bender J.K., Fleige C., Klare I., Fiedler S., Mischnik A., Mutters N.T., Dingle K.E., Werner G. (2016). Detection of a *cfr*(B) Variant in German *Enterococcus faecium* Clinical Isolates and the Impact on Linezolid Resistance in *Enterococcus* spp.. PLoS ONE.

[15] Cafini F., Nguyenle T.T., Higashide M., Roman F., Prieto J., Morikawa K. (2016). Horizontal gene transmission of the *cfr* gene to MRSA and *Enterococcus*: Role of *Staphylococcus epidermidis* as a reservoir and alternative pathway for the spread of linezolid resistance. J. Antimicrob. Chemother..

[16] Liu Y., Wang Y., Wu C., Shen Z., Schwarz S., Du X.D., Dai L., Zhang W., Zhang Q., Shen J. (2012). First report of the multidrug resistance gene *cfr* in *Enterococcus faecalis* of animal origin. Antimicrob. Agents Chemother..

[17] Diaz L., Kiratisin P., Mendes R.E., Panesso D., Singh K.V., Arias C.A. (2012). Transferable plasmid-mediated resistance to linezolid due to *cfr* in a human clinical isolate of *Enterococcus faecalis*. Antimicrob. Agents Chemother..

[18] LaMarre J.M., Howden B.P., Mankin A.S. (2011). Inactivation of the indigenous methyltransferase RlmN in *Staphylococcus aureus* increases linezolid resistance. Antimicrob. Agents Chemother..

[19] Sharkey L.K.R., O’Neill A.J. (2018). Antibiotic resistance ABC-F proteins: Bringing target protection into the limelight. ACS Infect. Dis..

[20] Wang Y., Zou Y., Xie J., Wang T., Zheng X., He H., Dong W., Xing J., Dong Y. (2015). Linezolid versus vancomycin for the treatment of suspected methicillin-resistant *Staphylococcus aureus* nosocomial pneumonia: A systematic review employing meta-analysis. Eur. J. Clin. Pharmacol..

[21] Brenciani A., Morroni G., Vincenzi C., Manso E., Mingoia M., Giovanetti E., Varaldo P.E. (2016). Detection in Italy of two clinical *Enterococcus faecium* isolates carrying both the oxazolidinone and phenicol resistance gene *optr*A and a silent multiresistance gene *cfr*. J. Antimicrob. Chemother..

[22] Cai J., Wang Y., Schwarz S., Lv H., Li Y., Liao K., Yu S., Zhao K., Gu D., Wang X. (2015). Enterococcal isolates carrying the novel oxazolidinone resistance gene *optr*A from hospitals in Zhejiang, Guangdong, and Henan, China, 2010–2014. Clin. Microbiol. Infect..

[23] Cui L., Wang Y., Lv Y., Wang S., Song Y., Li Y., Liu J., Xue F., Yang W., Zhang J. (2016). Nationwide Surveillance of Novel Oxazolidinone Resistance Gene *optr*A in *Enterococcus* Isolates in China from 2004 to 2014. Antimicrob. Agents Chemother..

[24] He T., Shen Y., Schwarz S., Cai J., Lv Y., Li J., Fessler A.T., Zhang R., Wu C., Shen J. (2016). Genetic environment of the transferable oxazolidinone/phenicol resistance gene *optr*A in *Enterococcus faecalis* isolates of human and animal origin. J. Antimicrob. Chemother..

[25] Huang J., Chen L., Wu Z., Wang L. (2017). Retrospective analysis of genome sequences revealed the wide dissemination of *optr*A in Gram-positive bacteria. J. Antimicrob. Chemother..

[26] Li D., Wang Y., Schwarz S., Cai J., Fan R., Li J., Fessler A.T., Zhang R., Wu C., Shen J. (2016). Co-location of the oxazolidinone resistance genes *optr*A and *cfr* on a multiresistance plasmid from *Staphylococcus sciuri*. J. Antimicrob. Chemother..

[27] Wang Y., Lv Y., Cai J., Schwarz S., Cui L., Hu Z., Zhang R., Li J., Zhao Q., He T. (2015). A novel gene, *optr*A, that confers transferable resistance to oxazolidinones and phenicols and its presence in *Enterococcus faecalis* and *Enterococcus faecium* of human and animal origin. J. Antimicrob. Chemother..

[28] Cavaco L.M., Korsgaard H., Kaas R.S., Seyfarth A.M., Leekitcharoenphon P., Hendriksen R.S. (2017). First detection of linezolid resistance due to the *optr*A gene in enterococci isolated from food products in Denmark. J. Glob. Antimicrob. Resist..

[29] Antonelli A., D’Andrea M.M., Brenciani A., Galeotti C.L., Morroni G., Pollini S., Varaldo P.E., Rossolini G.M. (2018). Characterization of *poxt*A, a novel phenicol–oxazolidinone–tetracycline resistance gene from an MRSA of clinical origin. J. Antimicrob. Chemother..

[30] Clinical and Laboratory Standards Institute (2019). Performance Standards for Antimicrobial Susceptibility Testing: Twenty-Nineth Informational Supplement M100S-S29.

[31] Mališová L., Jakubů V., Pomorská K., Musílek M., Žemličková H. (2021). Spread of Linezolid-Resistant *Enterococcus* spp. in Human Clinical Isolates in the Czech Republic. Antibiotics.

[32] Bender J.K., Fleige C., Klare I., Werner G. (2019). Development of a multiplex-PCR to simultaneously detect acquired linezolid resistance genes *cfr*, *optr*A and *poxt*A in enterococci of clinical origin. J. Microbiol. Methods.

[33] Kehrenberg C., Schwarz S. (2006). Distribution of florfenicol resistance genes *fex*A and *cfr* among chloramphenicol-resistant *Staphylococcus isolates*. Antimicrob. Agents Chemother..

[34] Gawryszewska I., Żabicka D., Hryniewicz W., Sadowy E. (2017). Linezolid-resistant enterococci in Polish hospitals: Species, clonality and determinants of linezolid resistance. Eur. J. Clin. Microbiol. Infect. Dis..

[35] Zahedi Bialvaei A., Rahbar M., Yousefi M., Asgharzadeh M., Samadi Kafil H. (2017). Linezolid: A promising option in the treatment of Gram-positives. J. Antimicrob. Chemother..

[36] Chajęcka-Wierzchowska W., Zadernowska A., García-Solache M. (2020). Ready-to-eat dairy products as a source of multidrug-resistant *Enterococcus* strains: Phenotypic and genotypic characteristics. J. Dairy Sci..

[37] Chajęcka-Wierzchowska W., Zadernowska A., Zarzecka U., Zakrzewski A., Gajewska J. (2019). Enterococci from ready-to-eat food—Horizontal gene transfer of antibiotic resistance genes and genotypic characterization by PCR melting profile. J. Sci. Food Agric..

[38] Patel S.N., Memari N., Shahinas D., Toye B., Jamieson F.B., Farrell D.J. (2013). Linezolid resistance in *Enterococcus faecium* isolated in Ontario, Canada. Diagn. Microbiol. Infect. Dis..

[39] Tamang M.D., Moon D.C., Kim S.R., Kang H.Y., Lee K., Nam H.M., Jang G.C., Lee H.S., Jung S.C., Lim S.K. (2017). Detection of novel oxazolidinone and phenicol resistance gene *optr*A in enterococcal isolates from food animals and animal carcasses. Vet. Microbiol..

[40] Fioriti S., Morroni G., Coccitto S.N., Brenciani A., Antonelli A., Di Pilato V., Baccani I., Pollini S., Cucco L., Morelli A. (2020). Detection of Oxazolidinone Resistance Genes and Characterization of Genetic Environments in Enterococci of Swine Origin, Italy. Microorganisms.

[41] Mendes R.E., Deshpande L.M., Costello A.J., Farrell D.J. (2012). Molecular epidemiology of *Staphylococcus epidermidis* clinical isolates from U.S. hospitals. Antimicrob. Agents Chemother..

[42] Lei C.W., Kang Z.Z., Wu S.K., Chen Y.P., Kong L.H., Wang H.N. (2019). Detection of the phenicol-oxazolidinone-tetracycline resistance gene *poxt*A in *Enterococcus faecium* and *Enterococcus faecalis* of food-producing animal origin in China. J. Antimicrob. Chemother..

[43] Schwarz S., Zhang W., Du X.D., Krüger H., Feßler A.T., Ma S., Zhu Y., Wu C., Shen J., Wang Y. (2021). Mobile Oxazolidinone Resistance Genes in Gram-Positive and Gram-Negative Bacteria. Clin. Microb. Rev..

[44] Yoon S., Son S.H., Kim Y.B., Seo K.W., Lee Y.J. (2020). Molecular characteristics of *optr*A-carrying *Enterococcus faecalis* from chicken meat in South Korea. Poultry Sci..

[45] Zhang Y., Dong G., Li J., Chen L., Liu H., Bi W., Lu H., Zhou T. (2018). A high incidence and coexistence of multiresistance genes *cfr* and *optr*A among linezolid-resistant enterococci isolated from a teaching hospital in Wenzhou, China. Eur. J. Clin. Microbiol. Infect. Dis..

[46] Alonso M., Marín M., Iglesias C., Cercenado E., Bouza E., García de Viedma D. (2014). Rapid identification of linezolid resistance in *Enterococcus* spp. based on high-resolution melting analysis. J. Microbiol. Methods.

[47] Smith T.T., Tamma P.D., Do T.B., Dzintars K.E., Zhao Y., Cosgrove S.E., Avdic E. (2018). Prolonged linezolid use is associated with the development of linezolid-resistant *Enterococcus faecium*. Diagn. Microbiol. Infect. Dis..

